# Silicon confers protective effect against ginseng root rot by regulating sugar efflux into apoplast

**DOI:** 10.1038/s41598-019-54678-x

**Published:** 2019-12-03

**Authors:** Ragavendran Abbai, Yu-Jin Kim, Padmanaban Mohanan, Mohamed El-Agamy Farh, Ramya Mathiyalagan, Dong-Uk Yang, Suriyaprabha Rangaraj, Rajendran Venkatachalam, Yeon-Ju Kim, Deok-Chun Yang

**Affiliations:** 1 Graduate School of Biotechnology, College of Life Science, Kyung Hee University, Yongin, 446-701 South Korea; 2 Department of Oriental Medicinal Biotechnology, College of Life Science, Kyung Hee University, Yongin, 446-701 South Korea; 3 Centre for Nanoscience and Technology, K. S. Rangasamy College of Technology, Tiruchengode, 637215 Tamil Nadu India; 4 Dr. N.G.P Arts and Science College, Kalpatti road, Coimbatore, 641048 Tamil Nadu India

**Keywords:** Nanoparticles, Agricultural genetics, Microbe, Biotic

## Abstract

Root rot caused by *Ilyonectria mors-panacis* is a devastating fungal disease leading to defect in root quality and causes reduced yield during the perennial life cycle of *Panax ginseng* Meyer. This indicates the imperative need to understand the molecular basis of disease development and also to enhance tolerance against the fungus. With this idea, the protective effect of silicon (supplied as silica nanoparticles) in *P. ginseng* root rot pathosystem and its molecular mechanism was investigated in the current study. We have tested different concentrations of silicon (Si) to disease-infected ginseng and found that long term analysis (30 dpi) displayed a striking 50% reduction in disease severity index upon the treatment of Si. Expectedly, Si had no direct degradative effect against the pathogen. Instead, in infected roots it resulted in reduced expression of *PgSWEET* leading to regulated sugar efflux into apoplast and enhanced tolerance against *I. mors-panacis*. In addition, under diseased condition, both protopanaxadiol (PPD) and protopanaxatriol (PPT) type ginsenoside profile in roots were higher in Si treated plants. This is the first report indicating the protective role of Si in ginseng-root rot pathosystem, thereby uncovering novel features of ginseng mineral physiology and at the same time, enabling its usage to overcome root rot.

## Introduction

*Panax ginseng* Meyer is an Oriental medicinal adaptogen and ginsenosides are the major pharmacologically active components of ginseng, which is proved to be effective against various diseases^1^. It is perennial in nature and the transition from vegetative to reproductive phase occurs at the third year and the accumulation of ginsenosides in roots increases with age^2^. Ginseng root rot caused by the fungus, *Ilyonectria mors-panacis* is one of the devastating diseases which initially infects the root tip and then proceeds until the crown. In addition, replanting results in infection of new plants. Hence, there is an imperative need to design strategies to overcome ginseng root rot. Younger age (~2 years) of the plant, acidic soil (pH 5.5–6.0), soil temperature (18–20 °C), high iron content are the major factors that promote the occurrence of root rot^3,4^.

Pathogen invades a plant to acquire nutrients which are majorly sugars, to support their growth and replication^5^. Understanding the molecular signaling events during plant-pathogen interaction is of great importance to establish strategies to overcome the pathogen. The plant defense system initially detects the pathogen, followed by the activation of the appropriate signal cascades. The downstream defense responses especially the crucial role of hormonal pathways such as SA (Salicylic acid), JA (Jasmonic acid) and Ethylene (ET) mediated pathways are well established. Periodic global transcriptome analyses by RNAseq revealed that JA and ET are majorly activated in ginseng-root rot pathosystem^6^. Previously, JA had been demonstrated to influence the triterpenoid pathway in ginseng and *PgSE2* was found to influence phytosterol biosynthesis^7,8^. Phytosterols are essential component of the plasma membrane that determines its rigidity/fluidity. Certain pathogens have the ability to modify the composition of the phytosterols in the plasma membrane to alter nutrient efflux^9,10^.

However, the molecular basis of root rot development in ginseng remains elusive and has to be addressed to enhance tolerance against the fungal disease. Perennial life cycle of ginseng, lack of allelic variations and difficulty in regeneration makes genetic improvement for root rot tolerance challenging at the moment. Therefore, priming of defense responses is considered as one of the feasible options. In a recent study, *Bacillus amyloliquefaciens* AK-0 was found to be antagonistic to *Cylindrocarpon destructans* and decreased the disease severity index in 4-year-old ginseng^11^. However, its interaction with other rhizobacteria and its effect on younger aged ginseng (where root rot occurrence is predominant) is unknown. Considering the present scenario, the application of ‘protective agents’ that specifically induce plant defense responses upon pathogen invasion seems to be one of the promising strategies to overcome root rot.

The protective role of silicon in *Arabidopsis*-powdery mildew pathosystem is well documented. It has been confirmed to induce defense related genes and at the same time balance primary metabolism^12^. Similarly, over the years its stress responsive role has been demonstrated in many other scenarios as well^13–16^. Of late, Phytonanotechnology i.e. the application of nanoparticles for empowering agriculture, is of great interest. The uptake and translocation of mesoporous silica nanoparticles in maize is already demonstrated^17,18^. In addition, silica nanoparticles were found to be compatible in *A. thaliana* up to 1000 mg. L^−1^ ^19^.

Based on this background, it was hypothesized that “Si might have protective effect in ginseng-root rot pathosystem when supplied as silica nanoparticle”. A series of experiments involving phenotyping in the presence and absence of Si, expression profiling of potential candidate genes/metabolites was carried out to unravel the role Si against ginseng root rot and its molecular basis. Overall, this study provides novel insights into the molecular factors governing defense responsiveness of Si and also on mineral physiology in the ginseng root-rot pathosystem.

## Results

### The protective role of Si in P. ginseng-root rot pathosystem

Various treatments include mock (HC), non-infected +1 mM Si (H-Si1mM), non-infected +2 mM Si (H-Si2mM), infected without silicon supplementation (IS), infected +1 mM Si (I-Si1mM) and infected +2 mM Si (I-Si2mM).

**Figure 1:**
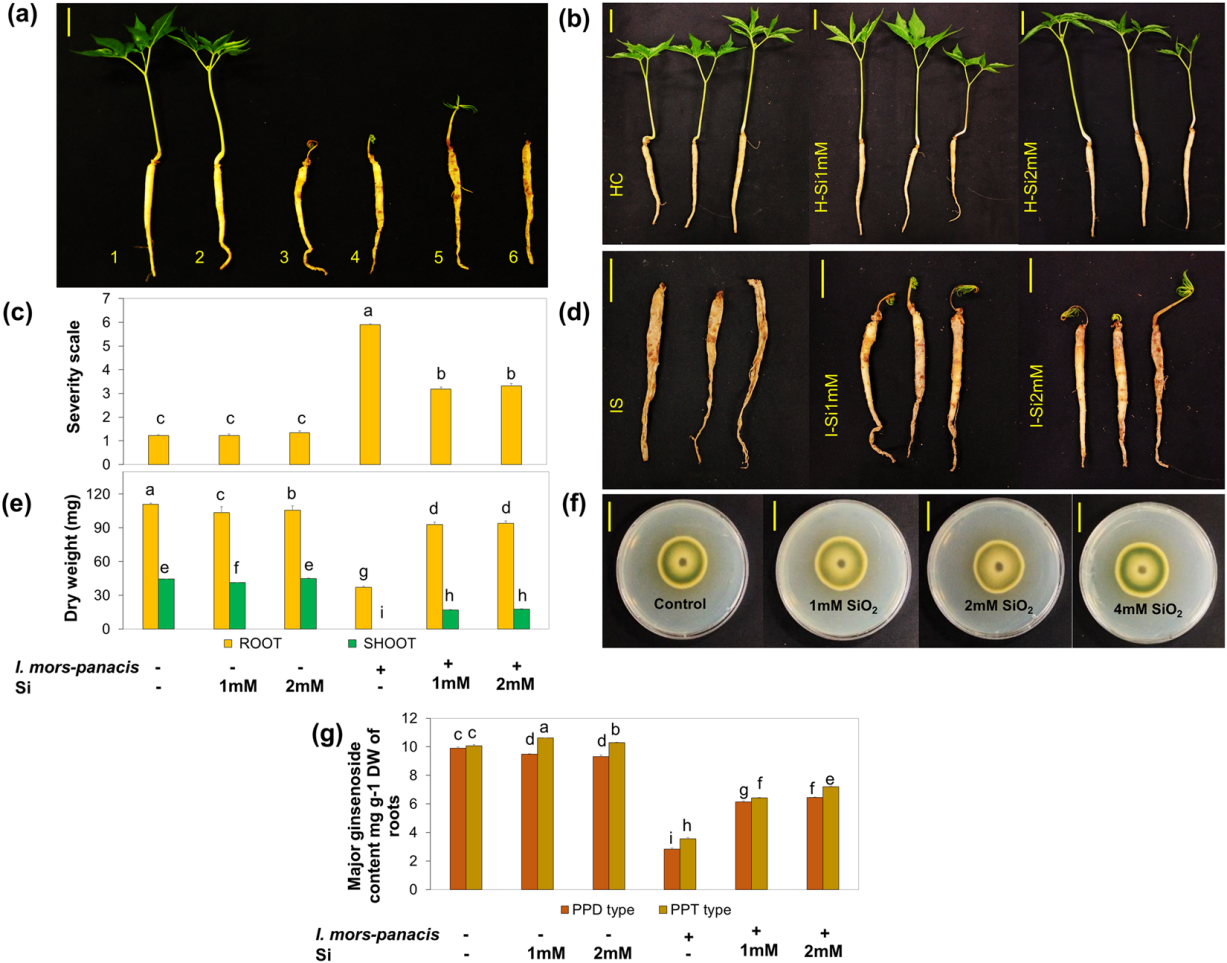
The protective effect of Si at Prolonged phase II. (**a**) Grading of plants into six categories on the basis of infection level in roots. (**b**) Non-infected seedlings did not show significant difference in phenotype due to Si supplementation. (**c**,**d**) Striking 50% reduction of disease severity index (DSI) in the Si treated seedlings was observed. No significant difference between the performance of 1 mM and 2 mM treatment of Si under infected conditions. (**e**) Si treatment reflected positive impact on Root and shoot biomass under pathogen infestation. (**f**) Silica nanoparticles did not display any direct degradative effect against *I. mors-panacis*. (**g**) Drastic decrease in total major ginsenoside profile was observed under infected conditions and interestingly a significant increase was documented in both PPD and PPT type of ginsenosides upon Si administration under infected conditions. Note: Scale: 2 cm; mock (HC), non-infected + 1 mM Si (H-Si1mM), non-infected + 2 mM Si (H-Si2mM), infected without silicon supplementation (IS), infected + 1 mM Si (I-Si1mM) and infected + 2 mM Si (I-Si2mM); PPD type: Rb1 + Rc + Rb2 + Rd; PPT type: Rg1 + Re + Rf.

At prolonged phase II (30 dpi), the seedlings were categorized into 6 grades based on the root phenotype and the disease severity index (DSI) was determined (Table S1; Fig. 1a). As expected, upon 1 mM and 2 mM administration of Si a striking 50% reduction in DSI was recorded, which confirms its protective effect against ginseng-root rot (Fig. 1b–d).

In addition, the shoot and root biomass were significantly improved in both I-Si1mM and I-Si2mM, than the IS (Fig. 1e). Furthermore, upon Si administration under infected conditions the total major ginsenosides in the root i.e., both PPD type (Rb1 + Rc + Rb2 + Rd) and PPT type (Rg1 + Re + Rf) were considerably improved in comparison with IS (Fig. 1g). However, in the absence of pathogen, Si treatment did not have any significant effect on the phenotype.

### Silica nanoparticles possesses no anti-fungal activity against *I. mors-panacis*

*In-vitro* screening was performed to examine the direct anti-fungal activity of silica nanoparticles against *I. mors-panacis*. Expectedly, it was found that the growth rate of the fungal pathogen was undisturbed even at 4 mM concentration (Figs. 1f and S1). This confirms that it does not have any direct anti-fungal activity which is consistent with previous reports in other plant species.

### Si regulates genes involved in Jasmonic acid (JA) signaling, triterpene, phytosterol biosynthesis and sugar efflux

**Figure 2:**
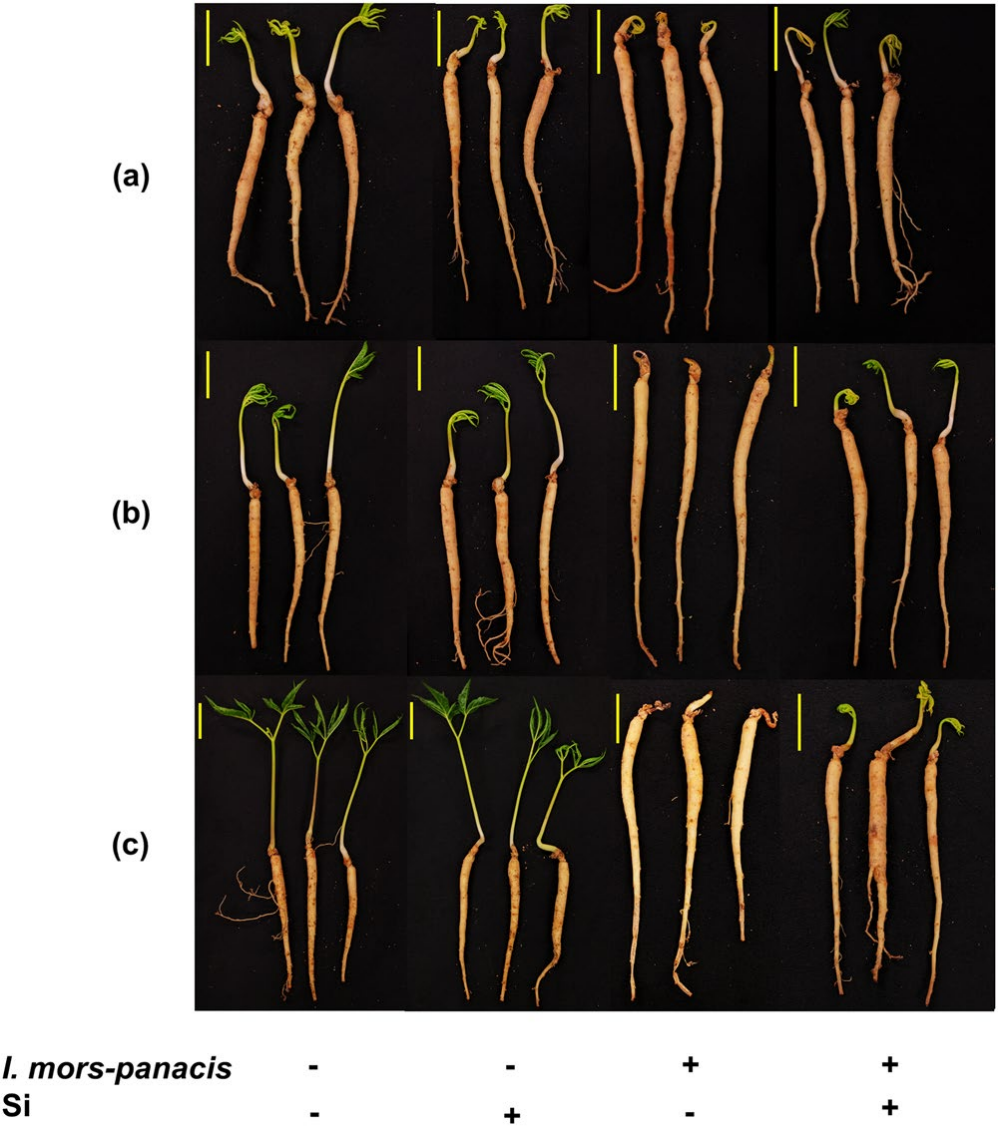
Time-dependent phenotypic analysis reveals the positive role of Si in enhancing the tolerance ginseng against the fungal pathogen. (**a**) At the early phase (4 dpi) the Si treated infected seedlings were completely similar to the mock. (**b**) During the intermediate phase (8 dpi) rotting initiated at the root tips of the infected seedling in the absence of Si, whereas, the Si supplemented seedlings were almost similar to the non-infected seedlings. (**c**) In the Prolonged phase I (16 dpi) severe infestation was observed in the absence of Si and on the other hand in the presence of Si only mild rotting symptoms were noted. Note: Scale: 2 cm; dpi: days post infection.

The phenotype of the seedlings at early phase (4 dpi), intermediate phase (8 dpi) and prolonged phase I (16 dpi) were observed prior to gene expression studies. Considerable improvement in terms of root rot tolerance was evident in each phase when Si was treated. Until the intermediate phase, I-Si1mM was almost similar to the non-infected seedlings with respect to shoot and root morphology (Fig. 2a,b). In addition, the phenotype of I-Si1mM in prolonged phase I was noticeably better than the IS (Fig. 2c).

Expression profiling of potential candidate genes was carried out using qRT-PCR. The expression of commonly used house-keeping genes such as *Pgactin*, *PgGAPDH* & *PgCYP* were evaluated and *PgGAPDH* was found to be ideal among them, hence was used as endogenous reference for profiling the target genes (Fig. S2).To understand the Si uptake into ginseng roots, silicon influx transporter, *Pglsi1* was identified from ginseng EST library based on homology analysis with orthologs of model plants. The expression of *Pglsi1* was induced in I-Si1mM, whereas it was significantly suppressed in IS (Fig. S3a). Under non-infected condition, when Si was treated, the expression of silicon influx transporter was unaltered.

To further determine the candidate pathways associated with biotic tolerance mediated by Si, the expression profile of major genes regulating JA biosynthesis and signaling, triterpene and phytosterol biosynthesis and sugar efflux pathways were analyzed. Further, the associated metabolites were also quantified. Considering that JA is one of key signaling molecule for defense response and also ginsenoside accumulation, JA biosynthesis and signaling related genes were investigated to trace out the molecular framework of Si mediated defense responses against the fungus. Initially, JA biosynthesis associated gene, *PgLOX6* (Fig. S3b) was profiled, and then the expression of JA signaling repressor, *PgTIFY10A* (Fig. S3c) was determined and finally Methyl Jasmonate was quantified (Figs. S4 and S5). These suggested that Si induced JA biosynthesis and signaling in response to *I. mors-panacis* infestation.

Further, the probable potential candidate transcription factors (TFs) involved in JA mediated activation of ginsenoside biosynthesis such as *PgMYC2a*, *PgMYC2b*, *PgWRKY22* and *PgMYB3* were profiled. Interestingly, in I-Si1mM these TFs expression were found to be induced in stage specific manner. *PgMYC2b* was induced in early phase, *PgMYB3* & *PgWRKY22* up-regulated in the intermediate phase, whereas *PgMYC2a* was enhanced in prolonged phase I (Fig. S3d–g, Table S2). On the other hand, all the above TFs were suppressed in IS.

### Regulation of mevalonic acid pathway to induce phytosterol accumulation

Expression profile of the rate-limiting genes of the mevalonic acid pathway, namely, *PgHMGR1*, *PgHMGR2* and *PgSS1* (Fig. S3h–j) clearly indicated the activation of Mevalonic acid pathway in I-Si1mM. However, the expression of genes associated with ginsenoside pathway such as *PgSE1* & *PgDDS* (Fig. S3k,l) was unaltered, while *PgBAS* (Fig. S3m) was down-regulated in I-Si1mM. Conversely, in the IS treatment, all the above mentioned genes were significantly down-regulated.

**Figure 3:**
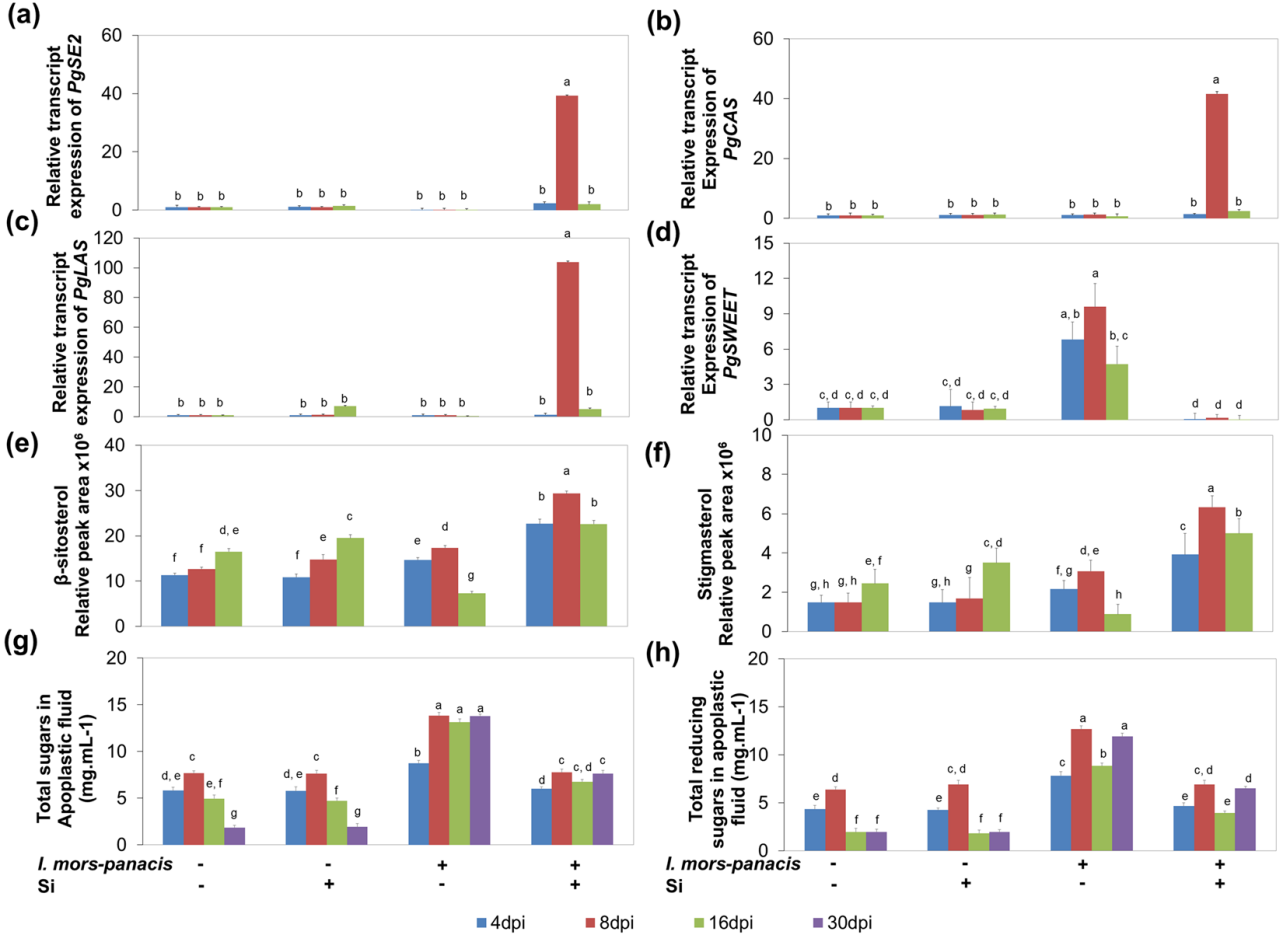
Si regulates sugar efflux into apoplast by increasing membrane sterols. Under infected condition, Si induces transcriptional re-programming leading to increased expression of (**a**) *PgSE2*, (**b**) *PgCAS*, (**c**) *PgLAS* and (**d**) reduced expression of the sugar efflux transporter, *PgSWEET*. Increased levels of (**e**) β-sitosterol and (**f**) stigmasterol confirms sterol accumulation and reduced profile of (**g**) total sugars, (**h**) total reducing sugars in the apoplastic fluid indicates regulated sugar efflux into apoplast upon Si treatment under infected conditions.

Next, the expression of key genes involved in the phytosterol biosynthesis such as *PgSE2*, *PgCAS* and *PgLAS* (Fig. 3a–c) was determined. Also, obtusifoliol 14α-demethylase, *PgCYP51* (Fig. S3n) was identified from ginseng EST library by homology search. The expression profile of these genes indicated the induction of sterol biosynthesis in I-Si1mM, whereas, it was severely down-regulated in the IS. Furthermore, the metabolite profile of β-sitosterol and stigmasterol was in line with the observed transcript abundance, thus confirming sterol accumulation in I-Si1mM and at the same indicating low sterol biosynthesis in IS (Figs. 3e and S4).

### Regulated sugar efflux into apoplast

Finally, the focus was on analyzing the sugar efflux into apoplast. Hence, sugar efflux transporter, *SWEET* (*Sugars Will Eventually be Exported Transporters*), which is important for sugar efflux into apoplast was identified in *P. ginseng* from the previously available EST library. The expression of *PgSWEET* was suppressed to a great extent during *P. ginseng*-*I. mors-panacis* interaction in the presence of Si (in I-Si1mM), which indicates the possibility regulated sugar efflux into apoplast. Contrastingly, in the IS, *PgSWEET* was significantly induced which sheds light on enhanced sugar efflux into apoplast (Fig. 3d).

Furthermore, the total and reducing sugars were profiled in root apoplastic fluid by anthrone and dinitrosalicalic acid DNS methods respectively. Expectedly, in I-Si1mM, the sugar content was almost similar to the non-infected samples, while, the sugar profile was significantly high in the IS (Fig. 3g,h). This clearly confirms that Si strictly regulates the sugar efflux into apoplast in ginseng-root rot pathosystem and on the other hand, the sugar efflux into apoplast is triggered in IS.

### Si regulates uptake of certain essential minerals

**Figure 4:**
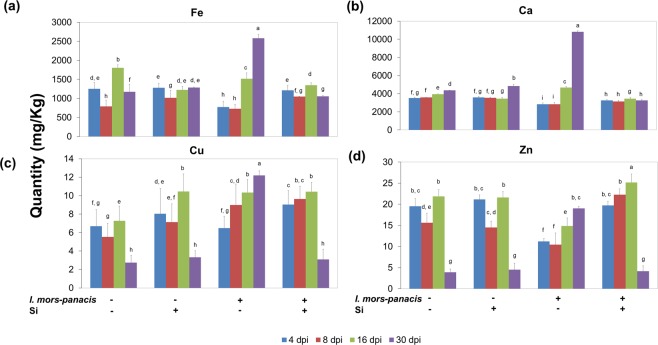
Si influences the uptake of candidate minerals in ginseng-root rot pathosystem. At prolonged phase II (30dpi) increased (**a**) Fe, (**b**) Ca, (**c**) Cu and (**d**) Zn profile was observed under infected condition when Si were not applied. On the other hand, upon Si administration during infected condition, the quantity of target minerals was similar to the mock.

The quantity of candidate minerals such as Iron (Fe), Calcium (Ca), Copper (Cu) and Zinc (Zn) were estimated in the experimental roots. The Ca content remained largely unaltered across all the treatments and time points, except in the IS at prolonged phase II, wherein Ca was significantly higher than all other treatments. Similarly, Fe, Cu and Zn profile of the IS at prolonged phase II was also significantly higher than any other treatments (Fig. 4). This suggests that there is a clear variation in the mineral uptake pattern between the I-Si1mM and the IS.

## Discussion

The protective role of silicon is well documented across several plant pathosystems^13–16,20^. Silicon is absorbed as silicic acid [Si(OH)_4_] by the plant system and silicon influx transporter, Lsi1 belongs to an aquaporin membrane protein family (Nod26-like major intrinsic protein (NIP) III which constitutively expresses in roots and coordinates the passive influx of silicic acid [Si(OH)_4_]^21,22^. But the mechanism of silica nanoparticles uptake into plants is not yet established. In the present study, *lsi1* was identified in ginseng and its up-regulation suggests that probably silica nanoparticles is somehow converted to silicic acid and then absorbed by the ginseng roots under infected condition. However, further confirmatory studies are essential to unravel the enigma associated with the uptake of Si into ginseng roots.

The total major ginsenoside content in roots was considerably higher in I-Si1mM at prolonged phase II as compared to IS. This indicates that ginsenosides might be the key players in overcoming the pathogen. Moreover, the plausible role of ginsenosides as defensive agents has been reported earlier^23,24^. Further, time course dependent global transcriptomic study revealed that majorly JA and ethylene (ET) mediated signaling is activated in ginseng-root rot pathosystem^6^. In another study, *PgLOX6* was characterized to be the precursor of JA biosynthesis in ginseng and *PgMYC*, *PgWRKY22* were indicated as the probable potential candidate TFs involved in JA mediated ginsenoside biosynthesis^7^. Besides, *PgMYB3* was found to be a JA responsive TF^25^. In the current study,  induction of *PgLOX6*, suppression of *PgTIFY10A* in I-Si1mM revealed the activation of JA biosynthesis and signaling. Metabolite profile of MeJA re-confirmed the above mentioned. Expression profile of the candidate JA signaling associated TFs indicated that *PgMYC2b* might be responsive in early phase, *PgMYB3* and *PgWRKY22* might be associated with intermediate phase and *PgMYC2a* might play a role in prolonged phase I.

In I-Si1mM, key upstream genes of Mevalonic acid pathway namely, *PgHMGR1*, *PgHMGR2* & *PgSS1* were upregulated. However, contradicting to our initial hypothesis, Si did not trigger the expression *PgSE1*, *PgDDS* & *PgBAS* and the ginsenoside biosynthesis cassette was not induced in response to *I. mors-panacis* infestation. Two *squalene epoxide* genes namely, *PgSE1* and *PgSE2*, former associated with ginsenoside biosynthesis and the later involved in the regulation of phytosterols production have been characterized in ginseng^8,26^. Therefore, *PgSE2* and other sterol biosynthesis associated genes such as *PgCAS*, *PgLAS*, *PgCYP51* were insevestigated and they were found to be significantly up-regulated in I-Si1mM. Further, the metabolic profile of β-sitosterol and stigmasterol confirmed that Si orchestrated sterol accumulation under Si supplmentation in root-rot pathosystem. Phytosterols, especially β-sitosterol, stigmasterol (membrane sterols) play a major role in regulating the orderliness of plasma membrane. Certain PAMPs (Pathogen Associated Molecular Patterns) like cryptogein have the capacity to specifically remove sterols from cell membrane thereby increasing its fluidity and thus resulting in easier nutrient acquisition^9^. In addition, the primary purpose of pathogen invading a plant is reported to be for obtaining nutrients, mostly sugars. Certain pathogenic fungi have the ability to manipulate the membrane bound sugar efflux transporter, SWEET to result in increased sugar efflux into apoplast, thereby supporting its growth and replication^27^. Recently, TAL effectors from *Xanthomonas citri* was reported to manipulate *GhSWEET10*, leading to bacterial blight in cotton^28^. Moreover, when stigmasterol is high, the nutrient efflux into apoplast was reported to be significantly reduced leading to tolerance against bacterial pathogens^10^. Similarly, in the current study *PgSWEET* and the sugar content in the apoplast were profiled to unravel the sugar efflux into apoplast. It was conclusive that sugar efflux into apoplast in I-Si1mM roots was strictly regulated and is the major factor contributing to the tolerance against root rot causing, *I. mors-panacis*.

Abundance of certain minerals during ginseng cultivation promotes the occurrence of root rot^4^. Foliar application of iron (Fe) has been correlated with the higher root rot occurrence in *Panax quinquefolius*. It was further demonstrated that Fe supports growth and sporulation of the fungus. At the infection site, the production of phenolic compounds increases the Fe levels, which is sequestered by the fungus, thereby promoting the occurrence of root rot^29^. In the present study, interestingly, mineral uptake pattern (Fe, Ca, Zn & Cu) in I-Si1mM was distinct from other treatments in early phase and intermediate phase, while, in the prolonged phase II the IS had a unique pattern of mineral uptake (Fig. S6). In regard with Fe, the IS had the highest quantity, thus adding on to its susceptibility, whereas its profile in I-Si1mM roots was similar to that of the non-infected roots, thereby increasing the tolerance towards the pathogen.

**Figure 5:**
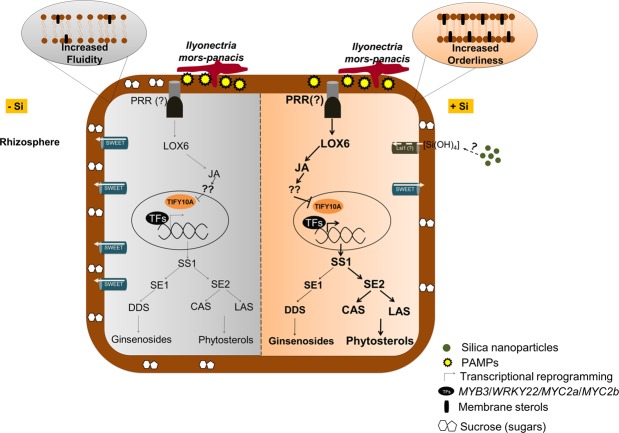
Transcriptional reprogramming mediated by Si leading to enhanced tolerance against *I. mors-panacis*. In the absence of Si higher expression of *PgSWEET* and reduced phytosterol profile mediated increased sugar efflux into apoplast, thus resulting in severe root rot. On the other hand, Si administration induced transcriptional reprogramming in ginseng roots, leading to regulated sugar efflux into apoplast via JA mediated sterol accumulation, thus enhanced tolerance against *I. mors-panacis*. Note: Jasmonic acid (JA); Transcription factors (TFs); Pattern Recognition Receptor (PRR).

Taken together, in IS the reduced phytosterols biosynthesis along with higher expression of *PgSWEET* enhanced the sugar efflux into apoplast, thereby supporting the growth and replication of the pathogen, resulting in severe root rot. Contrastingly, Si administration induced transcriptional reprogramming in ginseng roots, leading to regulated sugar efflux into apoplast via JA mediated sterol accumulation, thus enhanced tolerance against *I. mors-panacis* (Fig. 5). Furthermore, this is first study that comprehensively sheds light on the role of Si in modulating phytosterols accumulation and sugar efflux into apoplast under *I. mors-panacis* infection and at the same time reveals new insights into ginseng mineral physiology in root rot pathosystem.

## Conclusion

**Figure 6:**
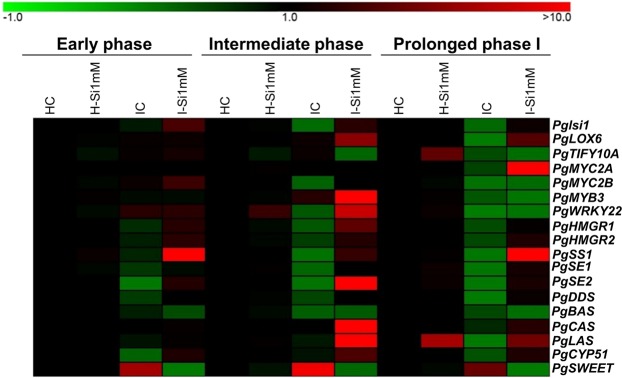
Heat map comprising of all the Si mediated ginseng root rot responsive genes determined in this study. These genes are believed to serve as potential candidates for genetic improvement of ginseng against root rot in the future.

The current study demonstrates the protective effect of Si against ginseng root rot. Interestingly, the root quality and major ginsenoside profile of I-Si1mM was much higher than the IS, hinting a positive impact in the market value. Expression and metabolite profiling indicated that Si regulated sugar efflux into apoplast via JA mediated phytostreol accumulation, thus leading to increased tolerance against *I. mors-panacis*. Additionally, the potential candidate root rot responsive genes determined in this study could be employed in molecular breeding programme and also in generating genetically engineered/edited lines to develop novel root rot tolerant ginseng varieties in the future (Fig. 6). Most importantly, Si treatment has the potential to address replant failure. In addition, the concentration of the pathogen used in this study is about 90 folds higher than the natural condition, which implies that Si treatment might have more pronounced effect in actual field. Taken together, the current study has open up the avenue for cultivating ginseng with enhanced tolerance against root rot.

## Materials and Methods

### Synthesis and characterization of silica nanoparticles

Silica nanoparticles were synthesized from rice husk using acid precipitation method followed by alkali extraction^17^. Rice husk was burnt at 750 °C using high temperature tubular muffle furnace for 3 h. The obtained rice husk ash (RHA) was treated with 6 N hydrochloric acid (HCl) under stirring conditions at 70 °C for 30 min to leach out impurities under acidic conditions. Then, the ash was washed with distilled water thrice to attain the pH of 7.0 and 2.5 N sodium hydroxide (NaOH) solution was added under stirring conditions for 2 h at 80 °C to extract silicate from ash. Concentrated sulphuric acid (H_2_SO_4_) was gradually added drop wise to the supernatant containing the extracted sodium silicate (Na_2_SiO_3_) until reaching pH 2.5, where the solution becomes transparent white silica sol. Then, it was thoroughly washed with double distilled water to remove the sodium interference and consequently washed with ethanol. Finally, the silica powder (99.7% purity) was collected after calcination at 450 °C for 2 h. The silica nanoparticle size ranged from 20–40 nm with spherical morphology^17^. The chemical reactions which occurred during the extraction of silica nanoparticles is as follows,

$${\rm{Rice}}\,{\rm{husk}}\,{\rm{ash}}\,(\mathrm{RHA})+(6\,{\rm{N}})\,{\rm{HCl}}\,\mathop{\longrightarrow }\limits^{{\rm{Leaching}}}\,{\rm{RHA}}+{\rm{Metal}}\,{\rm{impurities}}$$

$${\rm{RHA}}+(2{\rm{.5}}\,{\rm{N}})\,{\rm{NaOH}}\,\mathop{\longrightarrow }\limits^{{\rm{Alkali}}\,{\rm{extraction}}}\,{{\rm{Na}}}_{2}{{\rm{SiO}}}_{3}+{{\rm{H}}}_{{\rm{2}}}{\rm{O}}$$

$${{\rm{Na}}}_{2}{{\rm{SiO}}}_{3}+{\rm{Con}}{{\rm{.H}}}_{2}{{\rm{SO}}}_{4}\mathop{\longrightarrow }\limits^{{\rm{Acid}}\,{\rm{precipitation}}}{\mathrm{Si}(\mathrm{OH})}_{4}+{{\rm{Na}}}_{2}{{\rm{SO}}}_{4}$$

$${\mathrm{Si}(\mathrm{OH})}_{4}(\mathrm{Wet})\,\mathop{\longrightarrow }\limits^{{\rm{Drying}}\,{\rm{at}}\,{{\rm{100}}}^{\circ }{\rm{C}}}{\mathrm{Si}(\mathrm{OH})}_{4}\,(\mathrm{Dry})$$

$${\mathrm{Si}(\mathrm{OH})}_{4}\,(\mathrm{Dry})\,\mathop{\longrightarrow }\limits^{{\rm{Calcination}}\,{\rm{at}}\,{{\rm{450}}}^{\circ }{\rm{C}}}{{\rm{SiO}}}_{2}+{{\rm{H}}}_{{\rm{2}}}{\rm{O}}$$

### Plant materials, infection and growth parameters

Root rot causing fungal pathogen, *Ilyonectria mors-panacis* was activated on Potato Dextrose Agar (PDA) and was incubated at 25 °C. Ten day old mycelia was cut into discs of equal diameter using cork-borer and was mass produced in a commercially available vegetable juice under shaking conditions (150 rpm, 25 °C, 15 days). The pathogen was harvested and infected in artificial soil (Vermiculite, Perlite and Peat Moss – 3:1:1), in line with previously optimized protocol^17^. Silica nanoparticles at two different concentrations namely, 1 mM and 2 mM were mixed directly in soil along with the pathogen. Finally, the two-year-old homogenous ginseng seedlings were planted in pots and were cultured under controlled conditions (22 ± 2 °C, 12 h light, RH: 50 ± 2%). Mock (HC), non-infected 1 mM Si (H-Si1mM), non-infected 2 mM Si (H-Si2mM), infected without silicon supplementation (IS), infected 1 mM Si (I-Si1mM) and infected 2 mM Si (I-Si2mM) were the different treatments.

### Evaluating the protective role of Si

A long term analysis for 30 dpi was conducted to evaluate the potential of Si treatment to overcome ginseng root rot. DSI was determined by grading the ginseng seedlings based on the root phenotype at 30 dpi. DSI was calculated using the equation: DSI = [(X1 × 1) + (X2 × 2) + (X3 × 3) + (X4 × 4) + (X5 × 5) + (X6 × 6)]/(X1 + X2 + X3 + X4 + X5 + X6), where X1, X2, X3, X4, X5, and X6 are the number of plants with severity scales of 1, 2, 3, 4, 5, and 6 respectively.

### Extraction and quantification of ginsenosides

Ginsenosides from the target samples were extracted and analyzed as described in a previous study^30^. Six freeze dried roots from all the treatments at prolonged phase II were powdered and extracted twice by refluxing with 80% methanol at 70 °C for 1 h. The extract was filtered and in turn evaporated in a rotary evaporator at 45 °C. The resultant residue was dissolved in distilled water and was fractionated with 20 mL of water-saturated *n*-butanol twice. The butanol layers were mixed and evaporated to obtain crude ginsenosides. It was re-dissolved in 1 mL of HPLC grade methanol, filtered through 0.2 µm filter and was subjected to high performance liquid chromatography (HPLC) analysis on a C18 column with water and acetonitrile as mobile phase. The major ginsenosides were quantified with a one-point curve method by employing authentic external ginsenoside standards, obtained from the Ginseng Bank, Kyung Hee University, Republic of Korea.

### *In-vitro* analysis

The sensitivity of *I. mors-panacis* to silica nanoparticles was analyzed by evaluating the mycelial diameter in PDA plates. The pathogen was activated as described earlier and ten days old mycelia was cut into discs of equal diameter, then one disk was placed at the center of each plate supplemented with four concentrations of silica nanoparticles namely, 1 mM, 2 mM and 4 mM. The growth of the fungus was also analyzed on PDA without any external supplementation of silica nanoparticles and was considered as control. The growth rate of *I. mors-panacis* under various treatments was determined by using the formula: 100 − [(dc − dt)/(dc) × 100], where dc = Mycelial diameter of control; dt = Mycelial diameter upon silica nanoparticlestreatment.

### RNA extraction and expression profiling of target genes

Seedlings from all the treatments were sampled at early phase, intermediate phase, prolonged phase I and flash frozen in liquid nitrogen (LN_2_) prior to RNA extraction, employing TRI Reagent^®^ (Molecular Research Center, Inc, USA) with minor modifications to the standard protocol. About 100 mg of frozen roots were ground in pre-chilled mortar in to fine powder using LN_2_. 1 mL of TRI Reagent was added immediately to the homogenized samples and was gently vortexed. The supernatant devoid of tissue debris were collected by centrifugation (13, 500 rpm, 4 °C for 5 min). Then, by chloroform extraction, DNA, proteins and other metabolites were precipitated, while the RNA remained in the aqueous phase. The total RNA was harvested by precipitating it from the aqueous phase using isopropanol. Exactly, 1 µg of DNase treated RNA were reverse transcribed to single stranded cDNA using RevertAid Minus M-MuLV Reverse Transcriptase according to the manufacturer’s instructions (Fermentas, US). Quantitative Real-time PCR was performed in a 10 µL reaction volume using iQ^TM^ SYBR^®^ Green Supermix in a 96 well plate. The thermal cycler conditions were, initial denaturation for 10 min at 95 °C, followed by 40 cycles of 95 °C for 10 s, annealing for 10 s and 72 °C for 20 s. The fluorescent product was perceived at the final step of each cycle. It was conducted in CFX connect Real-time PCR detection system (Bio-Rad) and comparative cycle threshold (C_t_) values were documented for the target transcripts. The primer details are compiled in Table. S3. The relative quantity of the target transcript with reference to mock (HC) was determined based on 2^−ΔΔ*Ct*^ method^31^.

### Extraction and profiling of methyl jasmonate

Roots from early, intermediate and prolonged I phases were flash frozen with LN_2_ after sampling. The frozen roots were homogenized in pre chilled mortar and 500 mg of the fine powder was used for phytohormone extraction as described previously with few modifications^31^. About 5 mL of extraction buffer, 2-proponol/H2O/concentrated HCl (2:1:0.002, vol/vol/vol) was added. After vortexing the mixture was maintained under shaking conditions at 4 °C for 30 min. Following it, 10 mL of dichloromethane was added and once again the mixture was incubated under similar shaking conditions for 30 min. Then, upon centrifugation (10,000 rpm for 10 min), two phases were formed, of which the lower phase is of interest and is evaporated at 40 °C. The samples were re-dissolved in 400 µL of HPLC grade methanol and filter sterilized before LC-ESI-MS/MS analyses^32^.

### Profiling of phytosterols by GC-MS

About 200 mg of freeze dried powdered roots from various treatments were extracted separately for two times with 1 mL of CHCl3: MeOH (7:3) at room temperature. 20 μg of 5α-Cholestane was employed as the internal standard. The extract upon drying in a rotary evaporator was saponified with 1.5 mL of MeOH and KOH (20% (v/v)) at 80 °C for 1 h, aimed to hydrolyze the sterol esters. Following it, about 1.5 mL of MeOH and 4N HCl each was added and incubated at 80 °C for 1 h. Then, this mixture was extracted thrice with 4 mL of hexane. The sterols got separated to the hexane layer, which was further evaporated to dryness. The resulting powder was subjected to trimethylsilylation with pyridine and N, O-bis (trimethylsilyl) trifluoroacetamide + 1% trimethyl chlorosilane (1:1) for 90 min at 37 °C. Finally, GC-MS analysis was performed using a gas chromatograph (6890 A; Agilent Technologies) with a DB-5 (MS) capillary column (30 m x 30.25 mm, 0.25-mm film thickness) coupled with a mass spectrometer (HP 5973MSD). The reaction conditions were exactly similar to^30^. The peak area ratios of molecular ions of the endogenous sterol and that of internal standard were considered to be the actual endogenous sterol levels.

### Investigation of sugar profile in the apoplastic fluid

The apoplastic fluid was extracted by following standard protocol^10^ with minor modifications. *Panax ginseng* roots were immersed in a beaker containing 1 L of autoclaved milliQ water and was subjected to vacuum for 5 min. Then, the roots were cut into pieces (horizontally) and then transferred to a 15-mL tube (with a hole punched in bottom) coupled with a 1.5 mL collection tube. The above mentioned set up was then inserted into a 50 mL template and was centrifuged at 5000 rpm for 15 min. The collected solution is the apoplastic fluid and was filter sterilized. Following it, the total and reducing sugars content was determined by Anthrone and DNS methods respectively.

### Quantifying target minerals

The target minerals namely, Iron (Fe), Zinc (Zn), Copper (Cu) and Calcium (Ca) were quantified by Inductively Coupled Plasma with a mass spectrophotometer (ICP-MS) in the ginseng roots across all treatments and time points (Early, Intermediate, Prolonged I and Prolonged II phases). The root samples were oven-dried, ground into powder and further acid digested (HNO_3_ and H_2_O_2_-1:4). Finally, the target minerals were profiled using ICP-MS.

### Statistical analysis

Standard error was calculated from three biological replications. The results were subjected to analysis of variance (ANOVA) by Duncan’s multiple comparison test analysis. Mean values represented by the same letter are not significantly different from each other, while, represented by different letters are significantly different.

## Supplementary information

Supplementary information
