# Supplementary material for: Silicon confers protective effect against ginseng root rot by regulating sugar efflux into apoplast
Source: Sci Rep. 2019 Dec 3;9:18259. doi: 10.1038/s41598-019-54678-x (PMC6890760; doi:10.1038/s41598-019-54678-x)
Supplement: Supplementary file 1 — Supplementary information [file 41598_2019_54678_MOESM1_ESM.pdf]

Silicon confers protective effect against ginseng root rot by regulating sugar efflux into apoplast

Ragavendran Abbai[a], Yu-Jin Kim[b], Padmanaban Mohanan[a], Mohamed El-Agamy Farh[a], Ramya Mathiyalagan[a], Dong-Uk Yang[b], Suriyaprabha Rangaraj[c], Rajendran Venkatachalam[c, d], Yeon-Ju Kim[b*], Deok-Chun Yang[a, b*]

[a]Graduate School of Biotechnology, College of Life Science, Kyung Hee University, Yongin 446-701, South Korea.
[b]Department of Oriental Medicinal Biotechnology, College of Life Science, Kyung Hee University, Yongin 446-701, South Korea.
[c]Centre for Nanoscience and Technology, K. S. Rangasamy College of Technology, Tiruchengode 637215, Tamil Nadu, India.
[d]Dr. N.G.P Arts and Science College, Dr. N.G.P. Kalpatti road, Coimbatore-641048, Tamil Nadu , India

Running title: Protective effect of Silicon against *I. mors-panacis*

Summary: Silicon (supplied as silica nanoparticles) enhanced tolerance of *Panax ginseng* agains t the root rot causing fungus, *I. mors-panacis* by regulating sugar efflux into apoplast via JA med iated sterol accumulation and hence can be used overcome ginseng root rot.

*To whom correspondence should be addressed.

Deok-Chun Yang
Tel: +82-31-201-2100; E-mail: dcyang@khu.ac.kr

Yeon-Ju Kim
E-mail: yeonjukim@khu.ac.kr

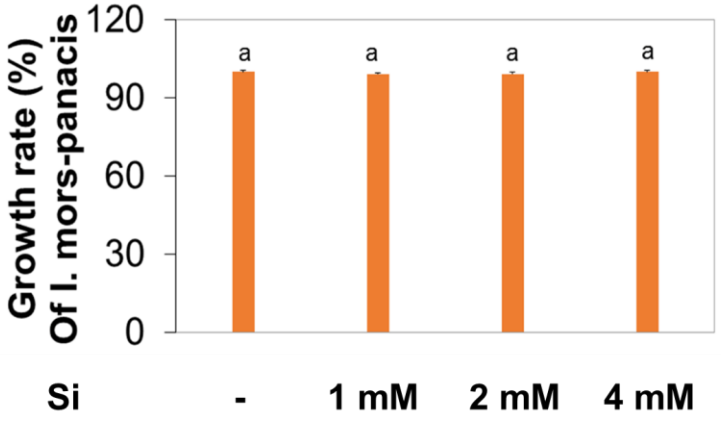

Fig. S1. In-vitro antifungal analysis of silica nanoparticles against *I. mors-panacis*. Fungal growth rate was analyzed on PDA plates with 1, 2 & 4mM concentrations of silica nanoparticles along with mock after incubation at 25°C for 10 days. There was no significant change in the growth rate of the pathogen even up to 4mM silica nanoparticles. This indicates that it does not possess any anti-fungal activity.

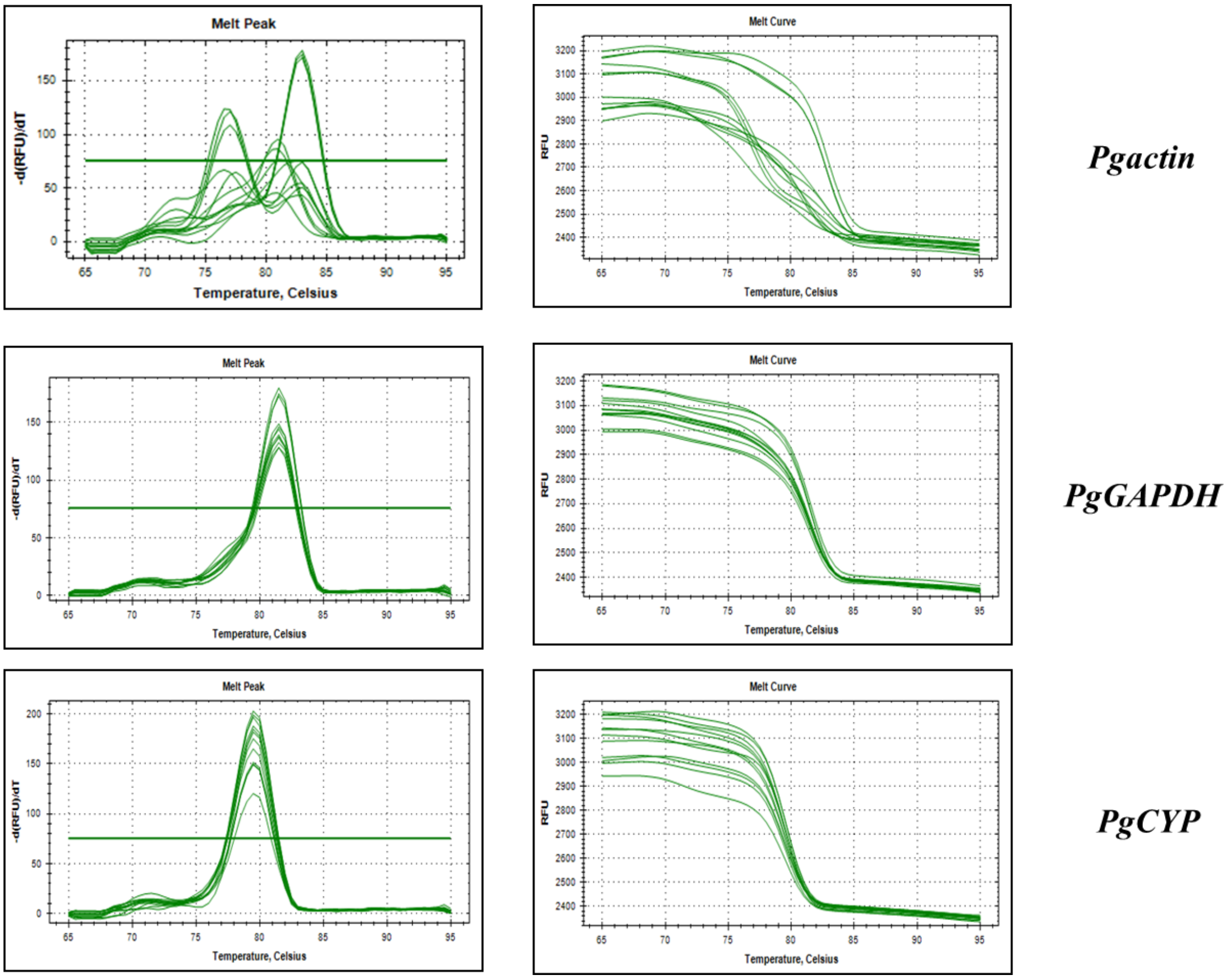

Fig. S2. Determination of the optimal house-keeping gene for qRT-PCR.

Among the analyzed three commonly used genes (*Pgactin*, *PgCYP* and *PgGAPDH)*, *PgGAPDH* was found to be the appropriate one based on the expression data.

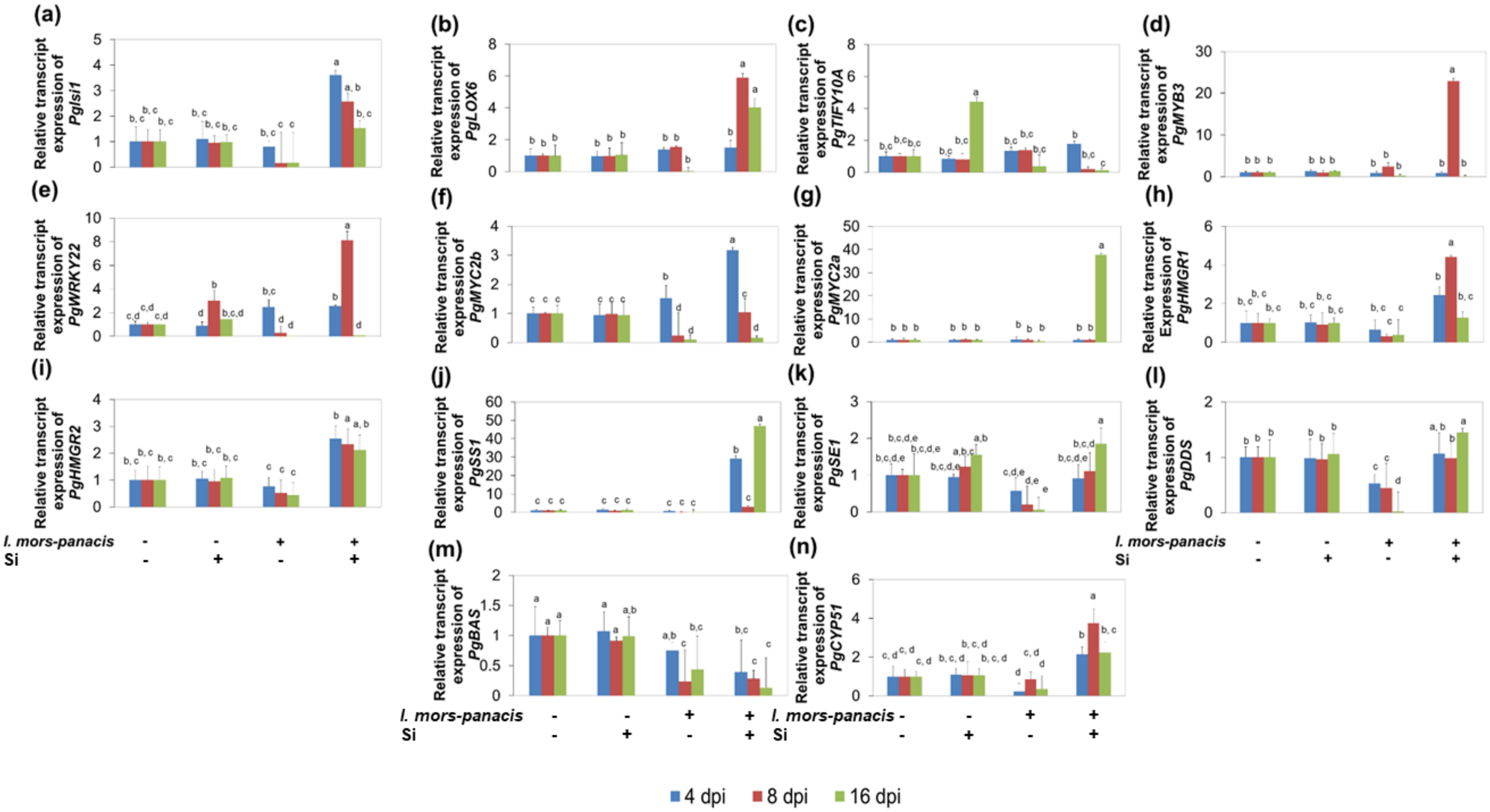

(a)
Relative transcript expression of PgIsi1
(b)
Relative transcript expression of PgLOX6
(c)
Relative transcript expression of PgTIFY10A
(d)
Relative transcript expression of PgMYB3
(e)
Relative transcript expression of PgWRKY22
(f)
Relative transcript expression of PgMYC2b
(g)
Relative transcript expression of PgMYC2a
(h)
Relative transcript Expression of PgHMGR1
(i)
Relative transcript expression of PgHMGR2
(j)
Relative transcript expression of PgSS1
(k)
Relative transcript expression of PgSE1
(l)
Relative transcript expression of PgDDS
(m)
Relative transcript expression of PgBAS
(n)
Relative transcript expression of PgCYP51
I. mors-panacis - - + +
Si - + - +
4 dpi
8 dpi
16 dpi

Fig. S3. Expression profile of the potential candidate genes via qRT-PCR. Firstly, the silicon influx transporter, (a) *Pglsi* was induced in I-Si1mM and on the other hand was suppressed in IS. In regard with genes associated with JA biosynthesis, the expression profile (b) *PgLOX6* and (c) *PgTIFY10A* indicated greater JA production in I-Si1mM as compared to IS. In addition, the TFs associated with JA signaling such as (d) *PgMYB3*, (e) *PgWRKY22*, (f) *PgMYC2b* and (g) *PgMYC 2a* showed stage specific induction in I-Si1mM. But, in IS all these TFs were suppressed. In the mevalonic acid pathway, upstream genes like (h) *PgHMGR1* and (i) *PgHMGR2* were up-regulate d in I-Si1mM and expectedly down-regulated in IS. The expression profile of ginsenoside biosyn thesis cassette (j) *PgSS1*, (k) *PgSE1* and (l) *PgDDS* along with (m) *PgBAS* and (n) *PgCYP51* indi cates the induction of phytosterol biosynthesis pathway in I-Si1mM.

Note: Jasmonic acid (JA); infected + 1mM Si (I-Si1mM); infected without Si supplementation (I S); Transcription factors (TFs)

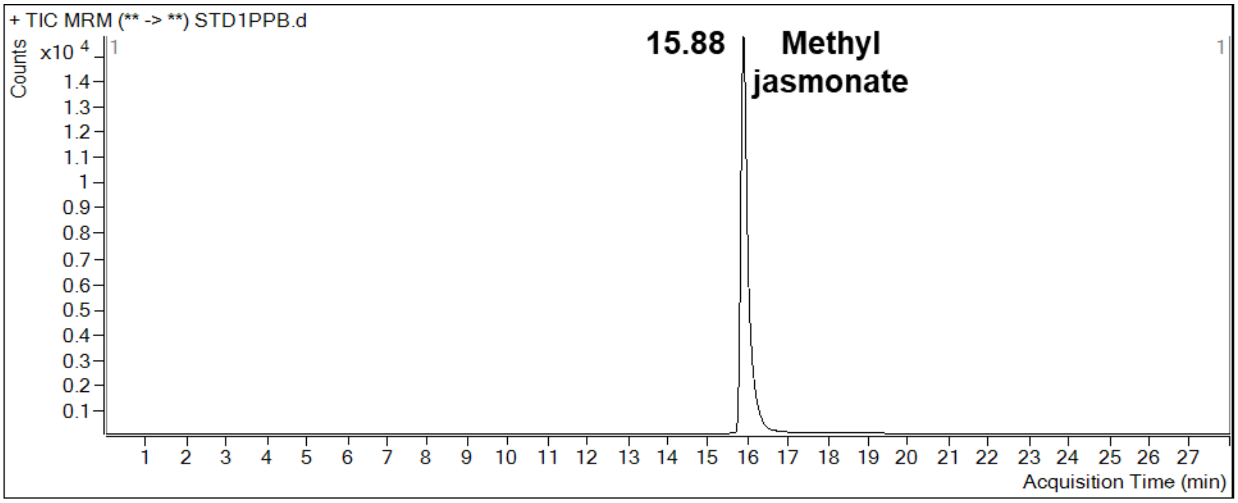

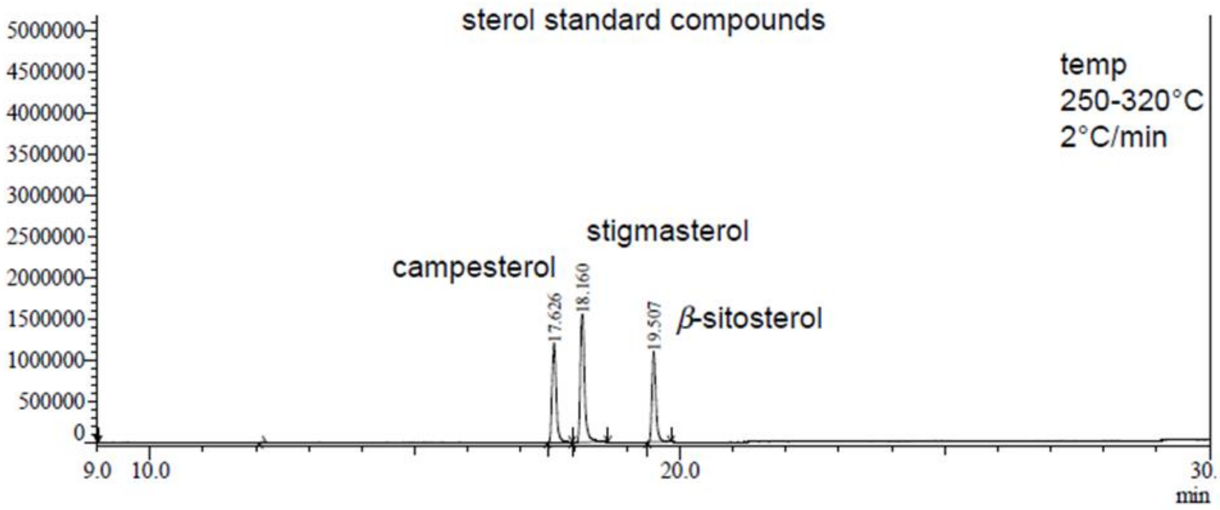

Fig. S4. Chromatograms of methyl jasmonate and phytosterol standards

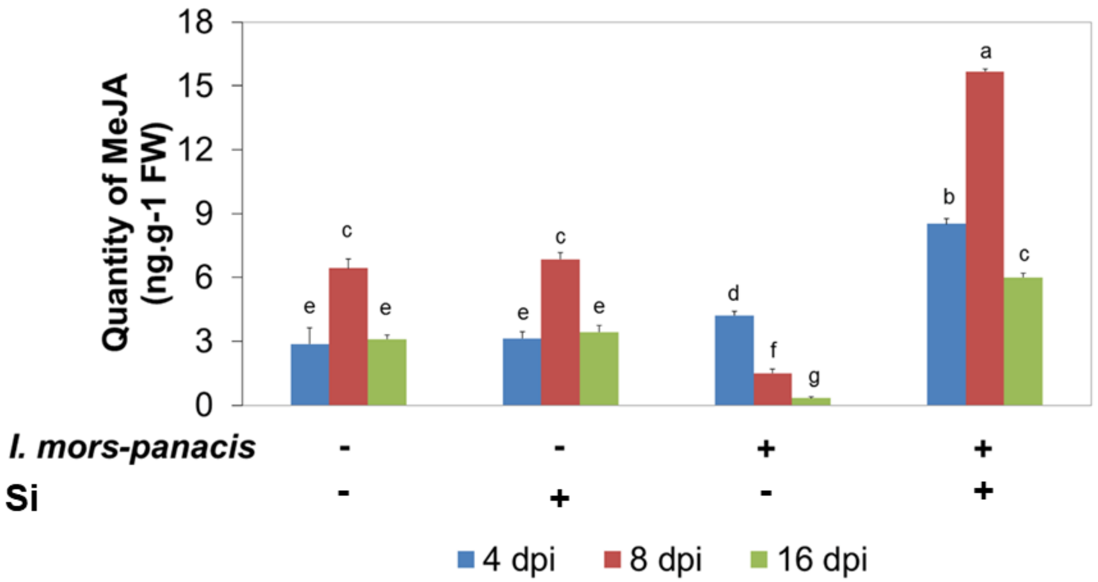

Fig. S5. Silicon induced MeJA in I-Si1mM. In line with the transcript abundance, in IS MeJA metabolite profile was suppressed and whereas in I-Si1mM it a significant increase was recorded.

Note: Methyl Jasmonate (MeJA); infected + 1mM Si (I-Si1mM); infected without Si supplementation (IS)

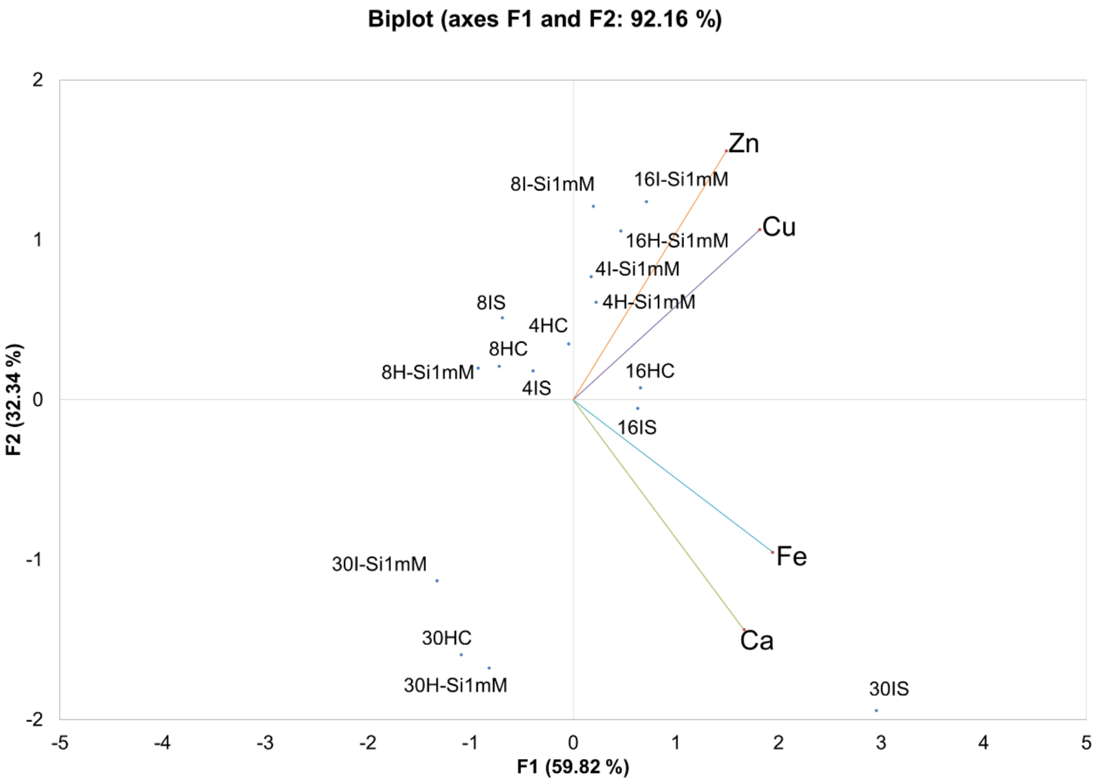

Fig. S6. Profile of potential candidate minerals across all the treatments in ginseng roots. PCA indicated a distinct mineral uptake pattern in IS during the prolonged phase II (30dpi).

Note: mock (HC), non-infected+1mM Si (H-Si1mM), infected without silicon supplementation (IS) and infected+1mM Si (I-Si1mM); The numbers 4, 8, 16 & 30, that precedes the sample indicates early, intermediate, prolonged phase I and II respectively. For instance, 8I-Si1mM indicates refers to the sample collected at 8dpi and the treatment is infected+ 1mM Si.

Table. S1. Description of scaling for Disease Severity Index (DSI)

Scale	Description
1	Roots are healthy with fine roots and well grown shoot
2	Healthy roots with well grown shoot
3	Bottom portion of the root is rotten and shoot is present
4	Half rotten root with shoot
5	Fully rotten root with shoot
6	Completely dead root without any shoot

Table. S2. Expression profile of potential transcription factors and their possible influence on silicon mediated protective role in ginseng root rot pathosystem

Gene	Function	Proposed role in current study
PgMYC2a	Candidates believed to be involved in JA mediated ginsenoside biosynthesis	Involved in silicon mediated str ess responsiveness during prolo nged phase 1 (16 dpi)
PgMYC2b		Involved in silicon mediated str ess responsiveness during early phase (4 dpi)
PgWRKY22		Involved in silicon mediated str ess responsiveness during inter mediate phase (8 dpi)
PgMYB3	JA signalling and responsiveness	Involved in silicon mediated str ess responsiveness during inter mediate phase (8 dpi)

Table. S3. List of primers used in this study

S. No	Gene	Primer sequence (5'-3')
**1**	*PgSS1*	FOR: ATGGGAAGTTTGGGGGCAATTCT<br>REV: GTTCTCACTGTTTGTTCAGTAGTAGGTT
**2**	*PgSE1*	FOR: AGCAGCAGTTGACAAAGG<br>REV: GCCACATTCGTTTTGGTGAAGG
**3**	*PgDDS*	FOR: ATGTGGAAGCTGAAGGTTGCTCAAGGA<br>REV: TTAAATTTTGAGCTGCTGGTGCTTAGGC
**4**	*PgLOX6*	FOR: TATACCCGGCTGGTTTTCTG<br>REV: ACTTTGTTCAATTCTAAATCCGAA
**5**	*PgTIFY10A*	FOR: GTCGGGCCAAATATCCACAC<br>REV: TTTTAGGCTCCATTGCTGCC
**6**	*PgWRKY22*	FOR: CCCACAAAGAAATCGTCGTT<br>REV: GGTACAAACCGGCACAAGTT
**7**	*PgMYB3*	FOR: TTCTCAAAGCTTGCCGACTT<br>REV: CCGGGTCCATGATAATTTTG
**8**	*PgSE2*	FOR: TTGCACCCCGGAGGATATTG<br>REV: AACTCTCCGGCCATCTTTGG
**9**	*PgCAS*	FOR: GGAGGAGATCATGGGGGTCT<br>REV: TCATGATGGCCCCTGGAGTA
**10**	*PgLAS*	FOR: CCAGTGCTTGTGCTTCAACC<br>REV: AAGCTTCCACATGCTACCCC
**11**	*PgBAS*	FOR: CTACCACCACACTCCGAAGG<br>REV: ACCAAGGGCGGAAGGAAAAA
